# Nondeterministic nature of sensorineural outcomes following noise trauma

**DOI:** 10.1242/bio.058696

**Published:** 2021-10-20

**Authors:** O'neil W. Guthrie, Ishan S. Bhatt

**Affiliations:** 1 Department of Communication Sciences & Disorders, Northern Arizona University, Flagstaff, AZ 86011, USA; 2 Cell & Molecular Pathology Laboratory, Northern Arizona University, Flagstaff, AZ 86011, USA; 3 Audiogenomics Research Laboratory, Department of Communication Sciences and Disorders, The University of Iowa, Iowa City, IA 52242, USA

**Keywords:** Sensory system, Hearing loss, Cochlea, Injury, Auditory

## Abstract

Over 1.1 billion individuals are at risk for noise induced hearing loss yet there is no accepted therapy. A long history of research has demonstrated that excessive noise exposure will kill outer hair cells (OHCs). Such observations have fueled the notion that dead OHCs underlie hearing loss. Therefore, previous and current therapeutic approaches are based on preventing the loss of OHCs. However, the relationship between OHC loss and hearing loss is at best a modest correlation. This suggests that in addition to the death of OHCs, other mechanisms may regulate the type and degree of hearing loss. In the current study, we tested the hypothesis that permanent noise-induced-hearing loss is consequent to additional mechanisms beyond the noise dose and the death of OHCs. Hooded male rats were randomly divided into noise and control groups. Morphological and physiological assessments were conducted on both groups. The combined results suggest that beyond OHC loss, the surviving cochlear elements shape sensorineural outcomes, which can be nondeterministic. These findings provide the basis for individualized ototherapeutics that manipulate surviving cellular elements in order to bias cochlear function towards normal hearing even in the presence of dead OHCs.

## Abstract (precis)

**Summary:** The current findings provide the basis for individualized ototherapeutics that manipulate surviving cellular elements in order to bias cochlear function towards normal hearing even in the presence of dead cells.

## INTRODUCTION

A fundamental concept in occupational and environmental medicine is the notion that excessive exposure to loud noise will kill outer hair cells (OHCs) in the cochlea which manifest as permanent noise induced hearing loss (NIHL) (Kirchner et al., 2012; Mirza et al., 2018). Histological analyses of human temporal bones have concluded that OHCs are the most vulnerable to noise induced cell death (Mcgill and Schuknecht, 1976). Such human observations combined with animal experiments provided the basis for the equal energy hypothesis, which suggests that the same average NIHL and degree of OHC death will develop after exposed to the same noise energy (Le et al., 2017; Ward et al., 1981). Numerous animal studies have confirmed this conclusion by demonstrating a relationship between noise exposure and cytocochleograms of dead OHCs or mean NIHL (Clark, 1991; Hamernik et al., 2007). The mechanisms by which noise exposure induces dead OHCs appear to be multiplicative and involve a variety of pathophysiological cascades (Kurabi et al., 2017; Sha and Schacht, 2017). As a consequence, a large number of pre-clinical ototherapeutics are focused on preventing the death of OHC as a necessary prerequisite to the prevention of NIHL (Kurabi et al., 2017; Lu et al., 2014; Lynch and Kil, 2005). Given that mammalian OHCs do not regenerate, these approaches are not only intuitive, but they engender hope for the millions of individuals who are at risk for NIHL.

Currently there is no ototherapeutic approach that has achieved enough success to be widely accepted. The persistent failure to produce a widely-accepted ototherapy is likely the result of a variety of factors (Sha and Schacht, 2017). However, the present paradigm in ototherapeutic research on NIHL is that the noise exposure kills OHCs and then the death of OHCs determine the magnitude and configuration of permanent NIHL (Murakoshi et al., 2015; Wang and Puel, 2018). Therefore, therapies are designed to limit pathophysiologic OHC processes in order to reduce OHC death and limit or prevent NIHL (Chen et al., 2020; Wu et al., 2020). This paradigm has enjoyed a long history and has provided useful information about OHC biology in general and their pathophysiology in particular. Yet, this information has not been successful in affecting clinical outcomes and there is still no widely accepted therapy to prevent or limit the development of NIHL. As a complement to this current paradigm, we propose an evolution in thinking by suggesting that future therapies may achieve some level of success by targeting and manipulating the remaining/surviving cochlear cells in order to influence the development of NIHL. Explicit to this line of thinking is the extreme notion that the characteristics of a given NIHL (e.g. severity of the loss) is more dependent on the remaining/surviving cellular elements of the cochlea rather than the missing/dead OHCs. In support of this line of thinking, consider that the relationship between OHC death and NIHL exhibits at best a modest correlation (Borg, 1987; Chen and Fechter, 2003; Clark and Bohne, 1978). For instance, areas of the mammalian cochlea with apparently normal OHC and regenerated synapses can, nonetheless, exhibit severe NIHL and other coding deficits (Chen and Fechter, 2003; Song et al., 2016). Drug ototoxicity research has also revealed that even profound loss of inner hair cells cannot consistently predict the degree and configuration of hearing loss (Lobarinas et al., 2013). Similarly, human temporal bone studies could not demonstrate an association between OHC loss and audiometric thresholds or between IHC loss and audiometric thresholds (Landegger et al., 2016). Somewhat similar findings have been reported in the avian inner ear, which is endowed with the capacity to regenerate hair cells and their synaptic structures. Here, regeneration of hair cells and their neural synaptic contacts in pigeons and chickens does not reliably lead to functional regeneration (Durham et al., 2000; Reng et al., 2001; Sun et al., 2001). Such examples provide a primordial indication that the preservation of OHCs and their synaptic elements does not necessarily equate to normal cochlear functions. Ultimately, the combined observations from mammalian and avian inner ears have raised the question of whether preservation of OHCs after noise exposure would consistently prevent NIHL. Furthermore, a systematic increase in noise level have been shown to not result in a corresponding increase in OHC death. For instance, cell death as a function of noise energy is not reliably predicted by the equal energy hypothesis (Erlandsson et al., 1980; Harding and Bohne, 2004). This suggests that NIHL is consequent to additional mechanisms beyond the noise dose and the death of OHCs.

In the current study, we attempt to interrogate the hypothesis that permanent NIHL is consequent to additional mechanisms beyond the noise dose and the death of OHCs. As a first-approximation, we expect to observe normal cochlear functions even in the presence of noise induced dead/missing OHCs; a surrogate indication that the loss of OHCs does not consistently result in permanent thresholds shifts. Furthermore, we expect that individual subjects who experience the same noise trauma and exhibit similar threshold loss would nonetheless possess cochleae that are functioning in independent ways; an indication that no two post-injury cochlea can be the same. This suggests that a single ototherapy may not be appropriate for individuals who experienced similar noise exposure and present with similar threshold loss. Indeed, this study provides the theoretical basis for the development of future individualized ototherapeutics that manipulate the surviving cellular elements in order to bias the injured cochlea towards normal (or near normal) threshold sensitivity.

## RESULTS

### Functional heterogeneity

**Fig. 1.**
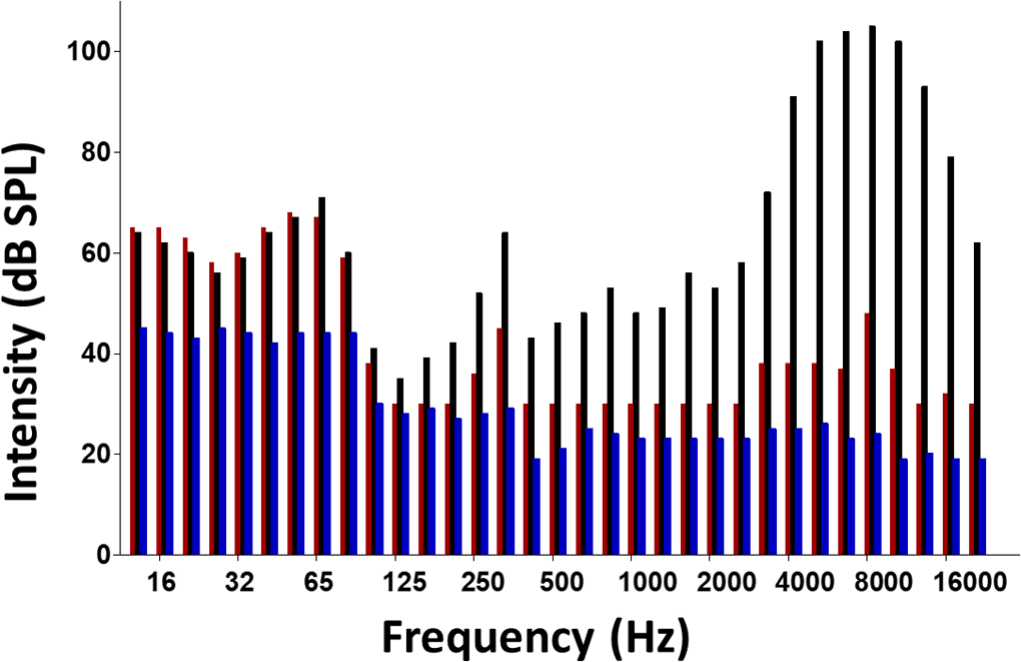
**Distribution of the hazardous noise energy used in the present study.** Noise energy was measured every hour during the 4 h of noise exposure. The noise energy was consistent for each hour during the 4 h of exposure. The red bars are the measured background noise with the hazardous noise energy turned off. The black bars are the hazardous (experimental) noise used in the current study. The blue bars are the measured background noise from the rat vivarium.

The working hypothesis is that permanent NIHL is consequent to additional mechanisms beyond the noise dose and the death of OHCs. If this is correct, then sensorineural outcomes will not be predictable from OHC loss and a given loss of OHCs will result in disparate functional outcomes. Sensorineural outcomes mean the test results from sensory testing [e.g. the use of distortion product otoacoustic emission (DPOAE) to test the function of sensory cells] and the results from both sensory and neural testing [e.g. the use of compound action potential (CAP) to test the function of both sensory cells and their neural elements]. Disparate functional outcomes refer to the various patterns of results (e.g. degrees of severity and loss configurations) from all functional tests. Therefore, we reasoned that a group of subjects exposed to the same traumatic noise dose should yield functional outcomes (DPOAE and CAP) that would manifest a wide variety of severity (e.g. normal to pathological) and configurations. Important to this line of thinking is the notion that all functional outcomes will be nondeterministic (unpredictable from the noise dose or OHC loss) and are expected to produce a variety of patterns (Mogensen and Malá, 2009; Overgaard and Mogensen, 2011; Wilms and Mogensen, 2011; Young et al., 2010). Fig. 3 reveals that exposure to the same loud noise can yield a variety of CAP threshold profiles. Fig. 3A shows CAP thresholds from a group of normal (non-exposed) animals while Fig. 3B shows CAP thresholds at 4 weeks after noise exposure from an experimental group of animals. Although all the animals were exposed to the same noise (Fig. 1), the heterogeneity in the threshold profiles from the experimental group is noteworthy. This suggests that the noise exposure alone may not predict the severity nor the configuration of threshold loss. Fig. 3C–F further illustrates this point by revealing contradictory threshold profiles from four individual subjects after they were exposed to the same noise dose. Among these animals, thresholds could range from normal to severely impaired and the thresholds adopted a variety of configurations. This suggests that each animal's cochlea has adopted a different functional outcome in response to the same noise exposure. Statistical testing further confirmed that there were significant threshold differences between the control and noise exposed groups [*t* (16)=1.676, *P*<0.05].

**Fig. 2.**
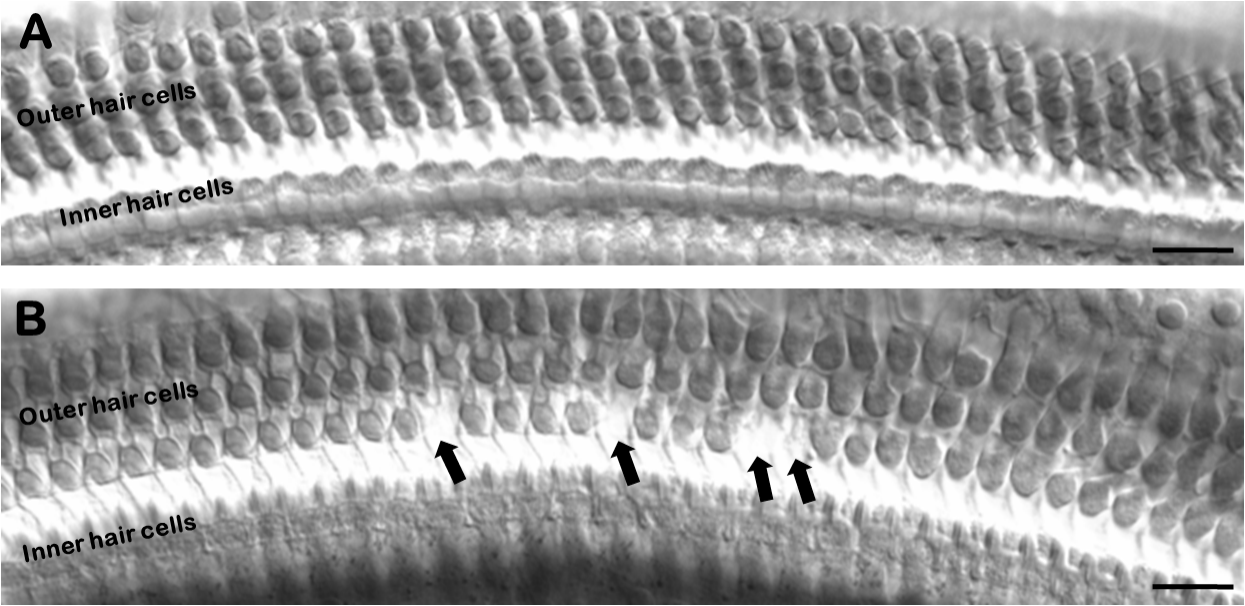
**Example of hair cell counts.** (A) Photomicrograph of the cochlear neurosensory epithelium with no loss of hair cells. (B) Photomicrograph of the cochlear neurosensory epithelium with four missing cells (see individual arrows). Scale bars: 100 µm.

**Fig. 3.**
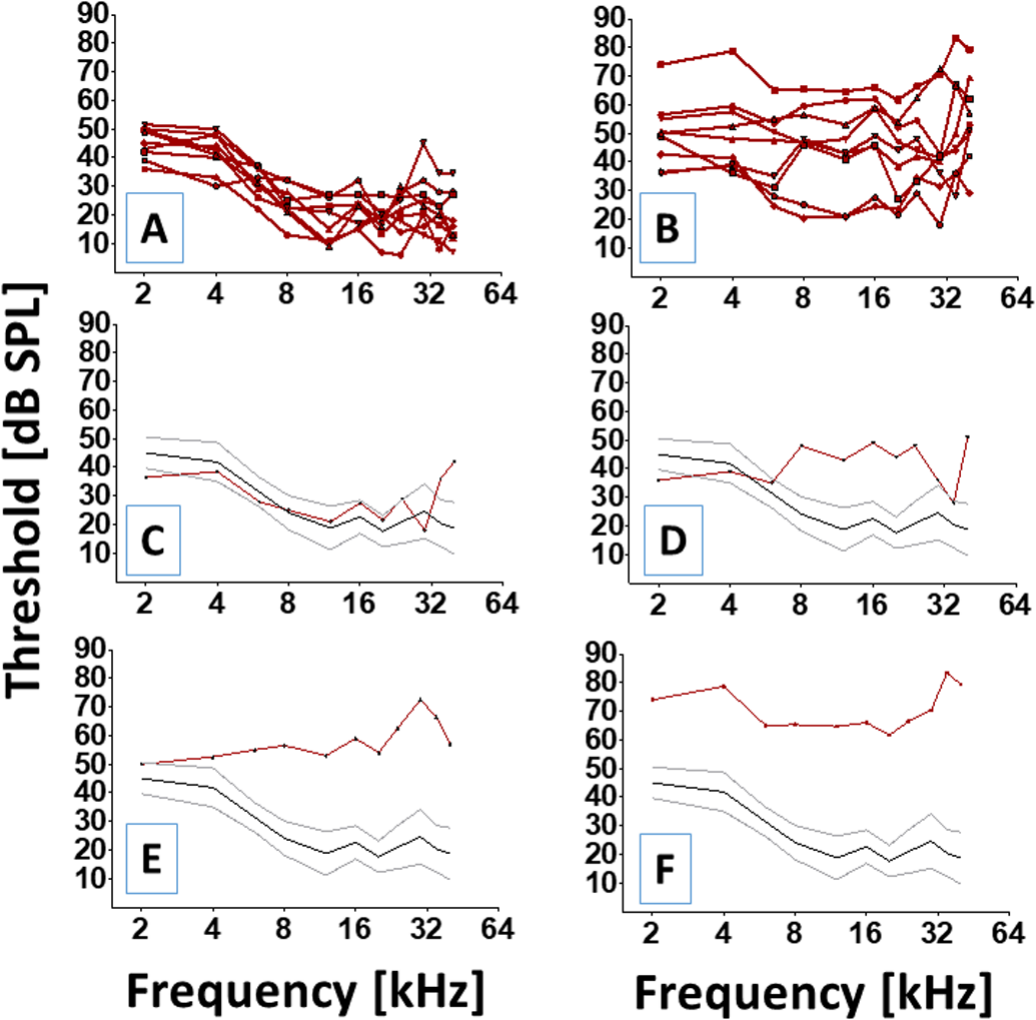
**Functional heterogeneity as revealed by CAP thresholds.** (A) Individual CAP thresholds (red lines and symbols) for each animal in the control group at 4 weeks. (B) Individual CAP thresholds for each animal in the noise exposed group at 4 weeks. Note the heterogeneity in threshold severity and configuration even though all the animals were exposed to the same hazardous noise. (C–F) CAP thresholds can manifest various levels (e.g. normal, or severely impaired) and configurations (e.g. high frequency or nearly flat) after exposure to the same hazardous noise. Red lines and symbols in each panel are CAP thresholds. The black continuous horizontal line is the mean threshold sensitivity for normal subjects and the accompanying gray continuous horizontal lines represent the upper and lower range for normal threshold sensitivity (one standard deviation above and below the mean).

**Fig. 4.**
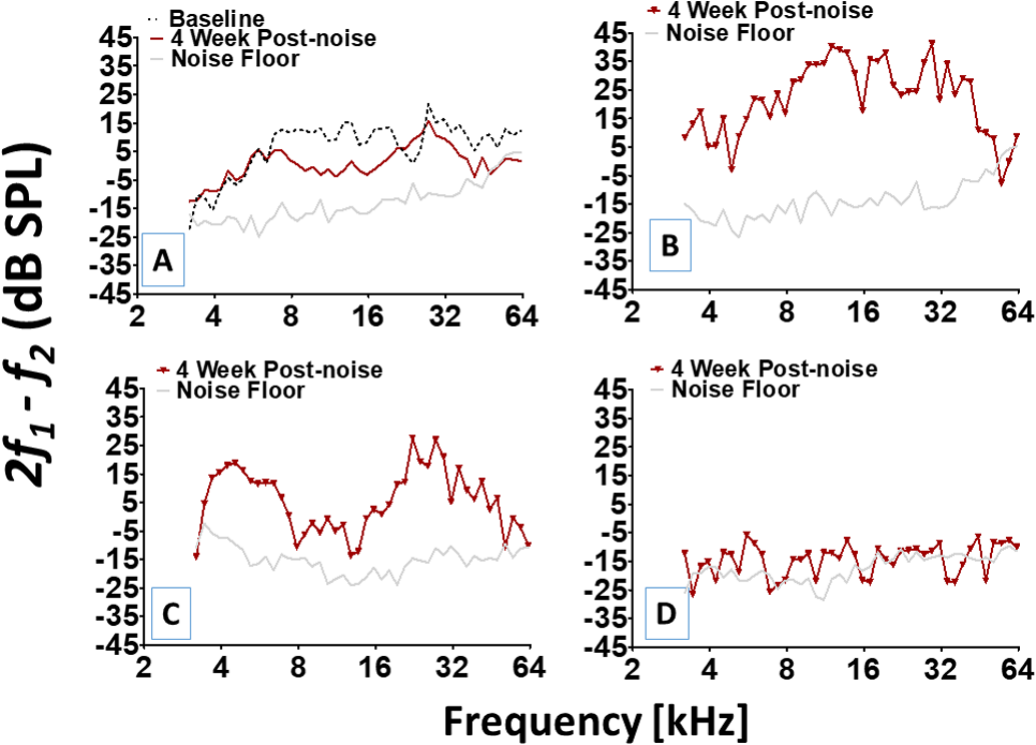
**Functional heterogeneity as revealed by DPOAEs.** (A) Mean DPOAE levels at baseline (black dotted lines) compared with mean DPOAE levels at 4 weeks post noise exposure from the same animals. There appears to be a permanent loss of DPOAE levels within the ∼8–16 kHz region. The lowest horizontal (gray) line is the measured noise floor and applies to all panels. (B–D) DPOAE levels from individual cochleae can manifest various forms (e.g. normal to severely depleted) and configurations (e.g. ‘cookie bite’ or nearly flat) after exposure to the same traumatic noise.

The DPOAE recordings in Fig. 4 provide additional independent support for the presence of differences in functional outcomes. Fig. 4A shows the mean DPOAE levels at baseline and at 4 weeks post-noise exposure from the same animals in Fig. 3B. Note the mean loss of DPOAE levels within the ∼8–16 kHz region, which would be expected given the power spectrum of the hazardous noise. However, when the mean data is decomposed and individual recordings are examined, it appears that each individual's cochlea is responding in unique ways. Fig. 4B–D reveals that individual cochleae can exhibit DPOAE responses that range from robust high-level responses (e.g. normal, Fig. 4B) to responses that are almost depleted into the noise floor (e.g. pathological, Fig. 4C,D). The DPOAE data are repeated measures, such that the same group of animals (noise exposed group) are measured before and after noise exposure. A repeated-measures ANOVA statistical computation revealed that there was a significant difference (*F*_[1,308]_=5.095, *P*<0.05) between DPOAE recordings made before and after noise exposure. The control group exhibited no loss of DPOAE and there were no significant differences in DPOAE recordings overtime.

### Cochlear individualism

**Fig. 5.**
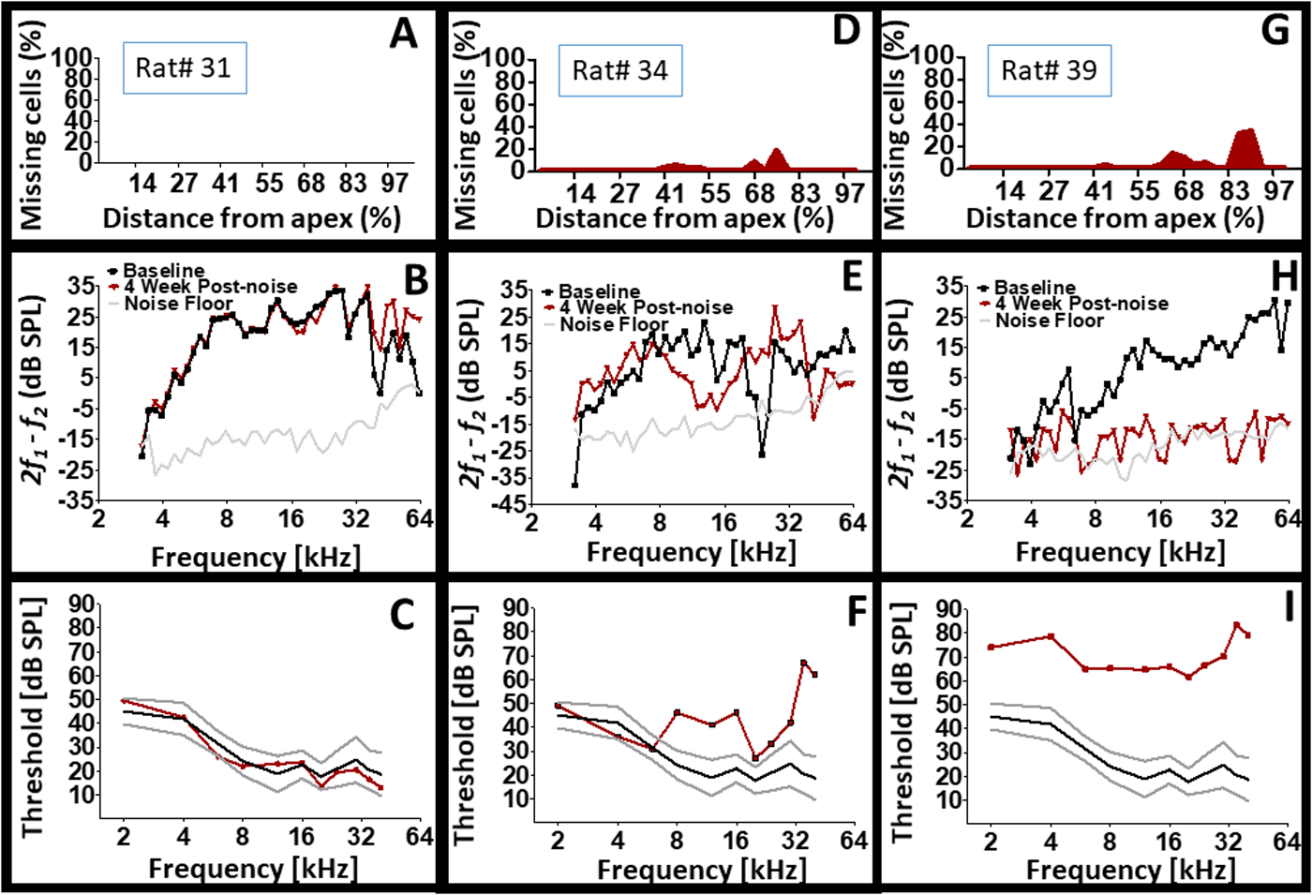
**Different degrees of injury after the same noise exposure.** (A–C) A control (normal) subject with no exposure to hazardous noise exhibited no dead outer hair cells (A); robust *2f_1_-f_2_* DPOAE (B) and normal CAP thresholds (C). (D–F) A subject exposed to noise who exhibited modest levels of OHC death (D), which was associated with modest levels of DPOAE loss (E) and modest levels of CAP threshold loss (F). (G–I) A subject exposed to the same noise who exhibited increased levels of OHC death (G), which was associated with increased levels of DPOAE loss (H) and increased levels of CAP threshold loss (I). Note that the two animals who were exposed to the same noise exhibited different structural and functional outcomes. Panels A, D and G are cytocochleograms of the percentage of missing (dead) OHCs as a function of percent distance along the cochlea from apex to base.

In other biological systems it is known that distinct structural alterations can nonetheless yield what appears to be similar functional outcomes (Kimble, 1992; Mogensen et al., 2007, 2008a,b). In further support of our hypothesis, we reasoned that no two post-injury cochleae can exhibit the same functional outcome (cochlear individualism); therefore, any two cochleae that appears to be the same on a single functional measure will nonetheless be distinct on another functional measure. Fig. 5 reveals a somewhat stereotypical outcome, where changes in OHC population and function is associated with changes in CAP threshold. Here, an increase in the level of dead OHCs is associated with increased loss of DPOAE levels and an increased loss of CAP thresholds. These outcomes are typical for animal studies that explore induced lesions to the cochlea (Pouyatos et al., 2002). Ordinarily, such outcomes are averaged across subjects, which helps to perpetuate what appears to be a strong association between pre- and post-synaptic functions following a given noise injury. However, a number of studies have revealed that averaging may lead to erroneous conclusions (Golowasch et al., 2002; Marder, 2011). Therefore, assessment of each individual from a sample (similar to individualized clinical assessments) may uncover patterns that would have been masked by averaging (Prinz, 2010). Although, Fig. 5 might be somewhat typical, it does support the notion of cochlear individualism, because the experimental animals were exposed to the same traumatic noise, yet their respective cochlear deficits are different.

**Fig. 6.**
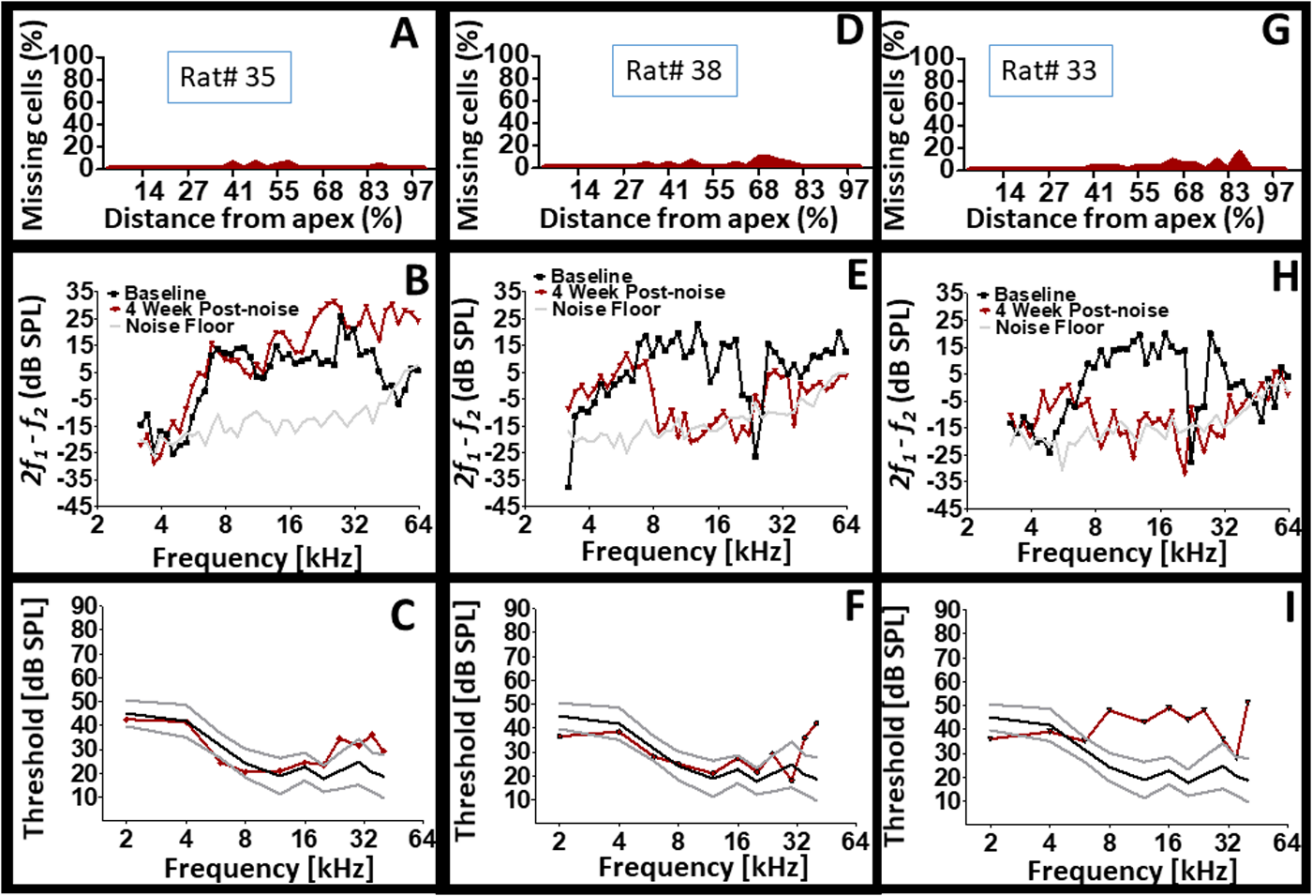
**Cochlear individualism.** (A,D,G) After noise trauma, modest (<20%) levels of dead OHCs were detected within the cochlea of three subjects. (C,F) Two of the three subjects exhibited similar (normal) CAP thresholds yet different types of DPOAE deficits. (E,H) Two of the three subjects exhibited similar DPOAE deficits yet different CAP thresholds.

**Fig. 7.**
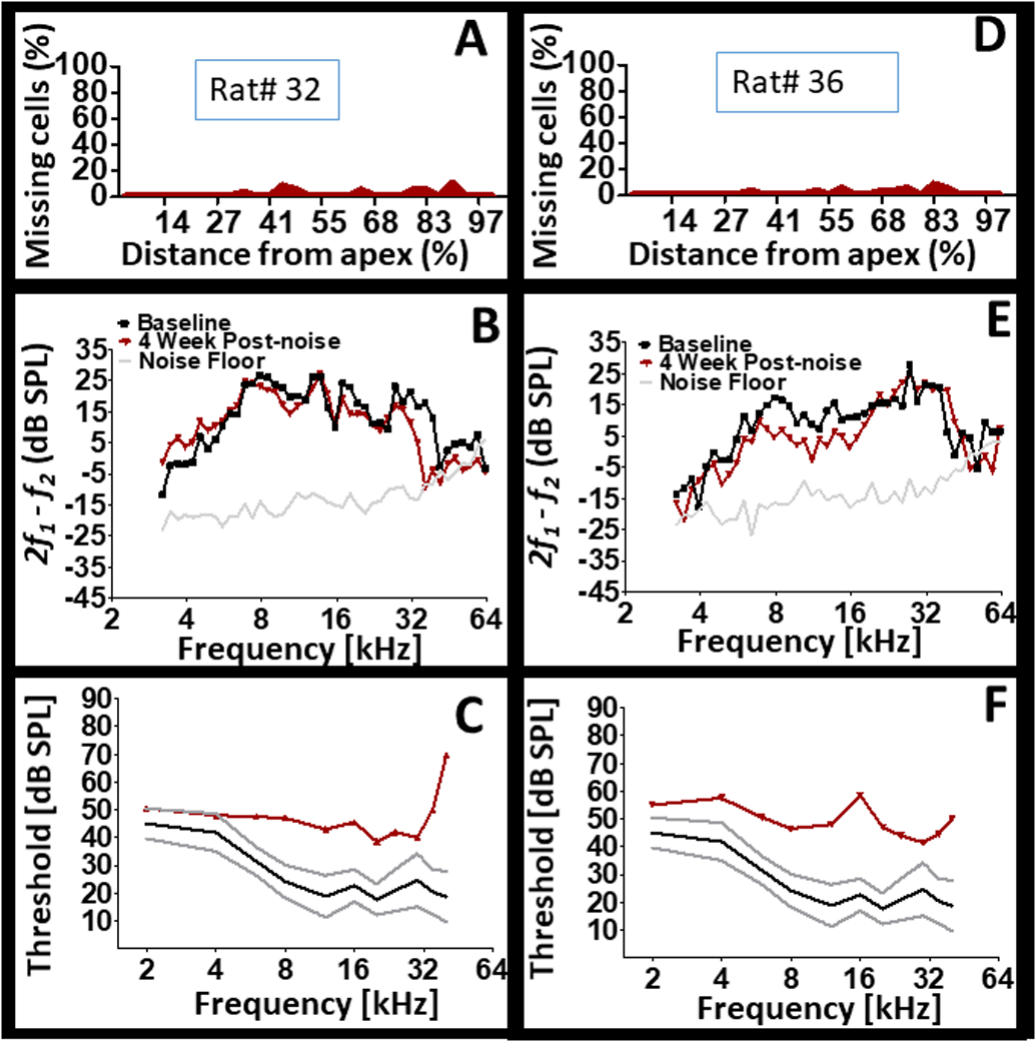
**Normal DPOAE with pathological CAP thresholds.** (A,D) After noise trauma, dead OHCs were detected within the cochlea of two subjects. (B,E) The two subjects exhibited largely normal DPOAE recordings. (C,F) The same two subjects also exhibited grossly pathological CAP thresholds.

Fig. 6 reveals that cochleae that appear to be the same may not actually be the same. An indication that plastic changes within the cochlea following noise exposure is specific to each animal's cochlea. The data for three individual subjects are depicted in Fig. 6. The cytocochleograms reveal the existence of dead OHCs in the cochlea of each subject, an indication that the noise dose was traumatic enough to kill a small proportion (<20%) of the cells. Two of the subjects demonstrated normal CAP thresholds over a wide range of stimulus frequencies. Therefore, based on the CAP thresholds of these two subjects, it would be facile to conclude that they were not affected by the noise exposure. However dead cells could be detected in both subjects and one subject exhibited a severe loss of DPOAE levels in the mid frequencies while the other exhibited improvement in DPOAE levels from mid to high frequencies. Therefore, neither the structural alteration (presence of dead OHCs) by itself, nor the DPOAE level by itself or CAP threshold alone would accurately reveal the functional status of each subject's cochlea. This is further illustrated by additional comparisons among the subjects in Fig. 6. Two subjects exhibited depleted DPOAE levels within the ∼8–24 kHz range. However, one subject exhibited normal CAP thresholds across a wide range of stimulus frequencies while the other subject exhibited abnormal CAP thresholds. If only the DPOAE levels were examined, then it would be facile to conclude that both subjects suffered from the same deficit since they each exhibited the same functional loss. In a clinical context both subjects would receive the same diagnosis and potentially the same treatment. However, if only the CAP thresholds were examined then it would be easy to conclude that one subject is physiologically normal while the other exhibits a pathology. In a clinical context, both subjects would receive different diagnoses and potentially different treatments (if any). Ultimately, the data demonstrates that there can be situations of incongruence between functional biomarkers of cochlear integrity following noise injury. Fig. 7 reveals the results for two additional subjects. The cytocochleograms demonstrated the existence of dead OHCs within the cochlea of both subjects. Interestingly, both subjects exhibited largely normal DPOAEs with pathological CAP thresholds. Therefore, what appears to be normal (in this case normal DPOAE) may not actually be normal. This conclusion is consistent with the data on CAP thresholds from Fig. 6C and F. Another important observation from these two animals, is the difference in threshold loss configuration even though DPOAE recordings across the same frequency range were largely normal.

**Fig. 8.**
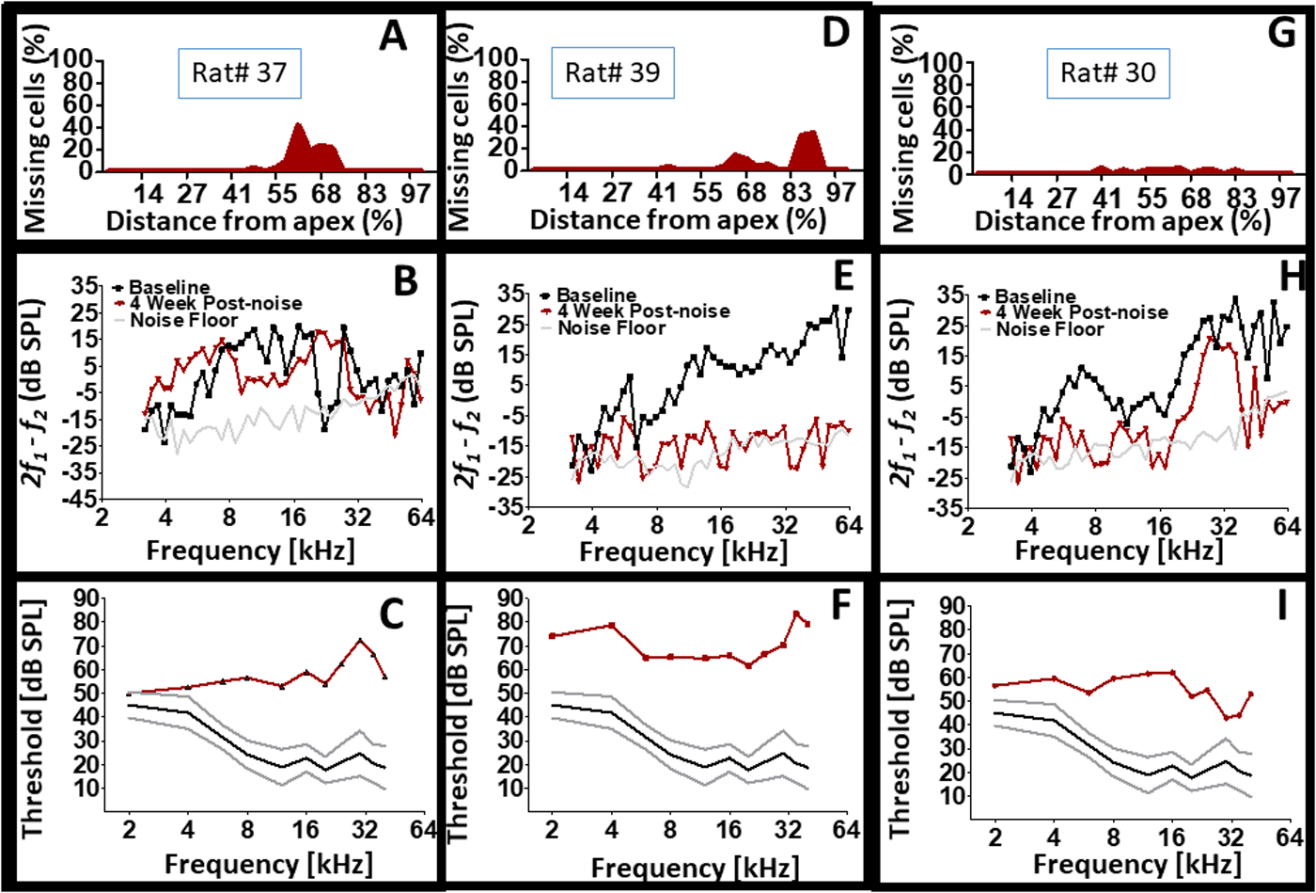
**Cochlear structural and functional heterogeneity after exposure to the same hazardous noise.** Note that pathological CAP thresholds are associated with different profiles of cell death (structure). Also, note that pathological CAP thresholds are associated with different types of DPOAE loss configurations. Panels A, D and G are cytocochleograms. Panels B, E and H are DPOAE recordings. Panels C, F and I are CAP thresholds.

Fig. 8 displays the results for three individual subjects. Interestingly, the level of dead OHCs appear inconsequential because both minor levels of cell death and more dramatic levels of cell death produced severe loss of CAP thresholds. Furthermore, the type of CAP threshold loss (e.g. low, mid, or high frequency loss) show no consistent association to the type of DPOAE loss or the location of dead cells. For instance, two subjects with dramatic levels of cell death produced different amounts and configurations of DPOAE and CAP losses. The combined results suggest that the presence of an injury within the cochlea may lead to various patterns of functional outcomes. The underlying basis for the above observations is unresolved. But it appears that the residual cellular elements within the cochlea are responding in different ways for each cochlea. For instance, a given cochlea may respond to injury by depleting sensorimotor functions (e.g. DPOAE) in favor of preserving normal sensorineural functions (e.g. CAP thresholds). Yet, another cochlea may respond to injury by depleting sensorineural functions in favor of preserving normal sensorimotor functions. Indeed, it appears that the presence of an injury can manifest a variety of functional outcomes.

Correlation analyses yielded results that were consistent with the findings described above. Table 1 displays Spearman correlation coefficients between all the experimental measures at 8, 16 and 30 kHz. These frequencies fell within the frequency band most affected by the noise dose (see Figs 3B and 4A). Interestingly, the level of dead cells associated with these frequencies along the basilar membrane showed at best a modest correlation with CAP or DPOAE loss at the same frequencies which is consistent with the results from other independent experiments (Borg, 1987; Chen and Fechter, 2003; Clark and Bohne, 1978).

**Table 1.**
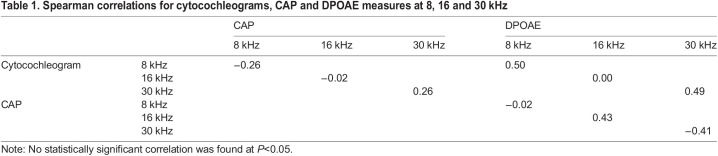
Spearman correlations for cytocochleograms, CAP and DPOAE measures at 8, 16 and 30 kHz

## DISCUSSION

Excessive exposure to noise is often considered a major determinant of hearing loss (Mirza et al., 2018). The average level of hearing loss tends to increase with the level or duration of the noise (Hong et al., 2013). Comprehensive noise exposure assessments that integrates both noise level and duration have revealed a linear association between average hearing loss and cumulative noise exposure (Lu et al., 2005). Explicit in such observations is the notion that a given hearing loss is directly dependent on the exposure (e.g. the exposure is the most relevant independent variable in the development of hearing loss). However, there are several studies that have found individuals who experience greater hearing loss than would be expected from their noise exposure while other studies have identified individuals who experience less hearing loss than would be expected from a given exposure (Davis et al., 2001; Erway et al., 1996; Hood, 1987; Irion, 1981; Li and Borg, 1993; Taylor et al., 1965). Furthermore, groups of individuals with similar noise exposures can exhibit a wide-range of hearing loss and individuals exposed to different noise exposures can develop similar hearing loss (Carlsson et al., 2005; Fortunato et al., 2004). Therefore, it appears that noise exposure may not be the only important variable that determines hearing loss (Li, 1992).

When demographic variables are held constant, there is some evidence that genetics might be a determining factor. Indeed, both human and animal studies have concluded that genotype is predictive of NIHL (Fairfield et al., 2005; Shen et al., 2014). However, humans with supposedly similar genotypes and similar exposures exhibit a wide range of NIHL (Carlsson et al., 2005). Mice studies have confirmed that subjects with similar genotypes can nonetheless exhibit a wide-variety of hearing loss including NIHL (Ingham et al., 2019; Myint et al., 2016). Therefore, it appears that both noise exposure and genotype are necessary, but neither is sufficient to explain variability in hearing thresholds following noise exposure (Carlsson et al., 2005). More direct factors that are often overlooked (yet influenced by both noise exposure and genotype), are the various cellular and subcellular changes which ultimately affect the physiological state of the organ (Guthrie, 2012, 2017; Guthrie and Xu, 2012; Xia et al., 2013). Such change in physiology is nondeterministic and expected to manifest various forms. Therefore, direct, or indirect measures of cochlear function should yield results that may range from normal to pathological or exhibit a large variety of pathological forms. This provides a basis to understand variability in hearing thresholds and other functional measures following noise exposure.

In the present study, animals exposed at the same time, to the same noise dose exhibited functional outcomes that were specific to each animal's cochlea. For instance, a given animal that presented with dead OHCs, may yield normal DPOAE with pathological CAP thresholds. Similarly, another animal may present with dead OHCs yet yield normal CAP thresholds and pathological DPOAE recordings. These examples of within subject incongruence in DPOAE and CAP profiles suggest that functional outcomes are not predictable from the noise exposure but instead specific to each cochlea. Between subject variability also provides some support for cochlear individualism. For instance, animals exposed to the same noise dose exhibited CAP thresholds that could be normal, moderately impaired, or severely impaired. Similarly, CAP threshold configuration exhibited a range of patterns within the noise exposed group. These results were confirmed by DPOAE recordings from the same animals. Here the DPOAE recordings also exhibited variable degrees of dysfunction and loss configuration.

The combined results from the current study suggest that sensorineural outcomes following noise exposure is nondeterministic and therefore does not support the equal-energy-hypothesis. This conclusion is directly orthogonal to that of previous research in the literature showing a deterministic relationship between hair cells loss and permanent threshold shifts. For instance, in noise-exposed chinchillas, threshold shifts that are greater than 5 dB has been linked to OHC loss (Hamernik et al., 1989). However, another study on noise-exposed chinchillas found that only threshold shifts greater than or equal to 35 dB is linked to OHC loss (Davis et al., 2004). Research on styrene ototoxicity among rats have shown that threshold shift does not occur with less than 33% loss of OHCs (Chen et al., 2008). However, a follow-up study using noise exposure showed that less than 20% loss of OHCs was linked to almost 30 dB threshold shift (Chen and Henderson, 2009). These studies when viewed individually show a deterministic relationship between threshold shift and a specific percent loss of OHCs. However, when view cumulatively, the inconsistent results from these studies further supports the notion that sensorineural outcomes following noise exposure is nondeterministic. In the current study, we show that OHC loss of less than 20% can result in a large variety of outcomes from normal to profound threshold elevations. The fact that such mild loss of OHCs (less than 20%) can result in various degrees and patterns of hearing loss provides further evidence that sensorineural outcomes following noise exposure is nondeterministic.

## MATERIALS AND METHODS

### Subjects

A total of 18 subjects (2-month-old, male, hooded Long-Evans rats) were used in the current study (Escabi et al., 2019; Holt et al., 2019; Ohlemiller, 2006). Epidemiological studies continue to demonstrate that NIHL is most prevalent among males, therefore we focused on male subjects in our study (Carroll et al., 2017). The subjects were acquired from Harlan Laboratories, Inc. (Livermore, CA, USA). They were then housed in an AAALAC (Association for Assessments and Accreditation of Laboratory Animal Care) accredited vivarium. The vivarium was approved and inspected by the United States Department of Agriculture yearly and by the Institutional Animal Care and Use Committee (IACUC) semi-annually. Background noise in the rat holding room of the vivarium was maintained at low sound pressure levels (SPL) even with cage washer and other equipment running at full capacity (≤50 dB SPL). All subjects had *ad libitum* access to food and water in environmentally enriched cages. Temperature was maintained at 21°C±1°C and a 12-h light/dark cycle was followed. Each subject was randomly assigned to a control (*N*=9) or experimental (noise exposed, *N*=9) group. Each group received baseline otoacoustic emission recordings under general anesthesia. Then the experimental group was exposed to noise. Four weeks after the noise exposure otoacoustic emission recordings were obtained again from both the experimental and the control groups. After these recordings, both groups underwent non-survival surgery in order to obtain action potential recordings and cytocochleograms. Subjects from both groups started and ended the study together. Before survival and non-survival procedures, the subjects received general anesthesia (ketamine/xylazine, 44/7 mg/kg). Atipamezole hydrochloride (Antisedan, 1 mg/ml) was used to facilitate quick recovery from the anesthesia following all survival procedures. All protocols regarding the use and handling of Long-Evans rats were evaluated and approved by the IACUC.

### Noise generation and exposure

The noise generation apparatus and procedure has been reported in detail previously (Guthrie, 2016; Yang and Guthrie, 2020). Briefly, broadband noise was driven by a DS335 Function Generator (Stanford Research System, Menlo Park, CA, USA) and band-pass filtered with a Frequency Device 9002-Dual-Channel Filter/Amplifier Instrument (Frequency Device Inc., Haverhill, MA, USA) to produce an energy band. This energy band was then amplified by a HCA1000A Parasound Amplifier (Parasound Products, Inc., San Francisco, CA, USA) and delivered to Vifa D25AG-05 speakers (Vifa International A/S, Videbaek, Denmark) located approximately 5 cm above the animals’ wire-cloth enclosure. Sound pressure measurements were made at the approximate level of the subject's pinnae using an OB-300Quest Type-1 Sound Pressure Meter (Quest Electronics, Oconomowoc, WI, USA). The distribution of sound pressure levels obtained at hourly intervals during the noise exposure is displayed in Fig. 1 (Feng et al., 2012; Liu et al., 2011). The duration of the noise was 4 h and the noise was an 8 kHz octave band.

The animals were conscious throughout the noise exposure epoch. They were staged in a wire-cloth enclosure (15×13×11 cm) that was placed within a 40 L reverberant cylindrical chamber. The noise was raised to 90-dB SPL; then, the animals were visually monitored for physical signs of stress (e.g. hyperactivity, excessive grooming, scrawling on the sides of the cage, etc.). After 1 or 2 min, the noise was slowly raised in 5-dB steps (at each step, the animals were visually monitored) until the desired noise level was reached. The desired SPL was 105 dB (linear settings), and this was measured with the OB-300Quest Type-1 meter at a level that approximated the rats’ pinnae. This particular sound pressure is known to induce dead outer hair cells along the basilar membrane of Long-Evans rats (Guthrie et al., 2011). The rats were exposed to the noise at the same time (10 am) and each rat was isolated in its own wire-cloth enclosure. Each rat was free to move within its enclosure and the sound intensity was verified at various locations.

### DPOAE

The DPOAE apparatus and protocol have been reported previously (Guthrie and Xu, 2012). Briefly, animals were ventrally positioned on a heated surgical table and their normal body temperature was maintained throughout the procedure. All DPOAE recordings were conducted in a sound attenuated booth. An ER-10B+emission probe assembly containing two speakers and a microphone was coupled to the external auditory meatus to produce the primaries (*F_1_* and *F_2_*) and record the *2f_1_-f_2_* DPOAE. The *F_2_/F_1_* frequency ratio was 1.25 and the corresponding level ratios was 1.18 (*L_1_/L_2_*), where *L_1_*=65 dB SPL and *L_2_*=55 dB SPL. A customized script written in LabVIEW (National Instruments, Austin, TX, USA) was used for presenting the primaries and acquire DPOAE recordings. A 0.2 cm^2^ hard-walled cavity that approximates the rat's external auditory meatus was used to calibrate the DPOAE recordings. These calibrations were free of artifacts and did not produce DPOAE SPLs that exceeded the noise floor.

### CAP thresholds

The CAP procedure is terminal (non-survival) and therefore deployed at the end of the study (4 weeks post-noise exposure). The procedure and apparatus are consistent with that of our previous work (Guthrie et al., 2011). Briefly, a ventrolateral surgical approach was deployed to open the auditory bulla. A silver-wire-recording electrode (A-M Systems, Inc., Carlsborg, WA, USA) with an outer-diameter of 0.1 mm was positioned on the surface of the round window membrane while a silver chloride electrode served as common. A SoundMax Integrated Digital Audio board was used in specifying stimulus parameters. The intensity of the stimulus followed a descending series in 1 dB steps. The lowest stimulus intensity needed to stimulate the cochlear nerve was recorded as threshold. Threshold was the lowest stimulus intensity in dB SPL needed to elicit a visually detectable neural response.

### Cytocochleograms

Cytocochleograms of missing OHCs were constructed for each animal as described in detail from our previous work (Guthrie et al., 2014, 2015). Briefly, this work was conducted at the end of the study (4 weeks post noise exposure) on the same animals that received CAP testing. Anesthetized animals were decapitated, and their cochleae were fixed *in toto* then washed with 0.1 M phosphate buffered saline and stained with 2% Osmium. Fig. 2 provides a representative example of how OHC loss was determined. The percentage of OHC loss as a function of distance from the apex of the cochlea was plotted for each animal. The Müller-rat frequency-place map was used to estimate frequency place along the length of the basilar membrane (Müller, 1991). This allowed for correlations between the number of dead hair cells at frequency specific areas of the cochlea with CAP threshold or DPOAE loss at the same frequency (Guthrie et al., 2011).

### Statistical analyses

Prism 5 version 5.03 (GraphPad Software, Inc., La Jolla, CA, USA) was used for statistical computations. The CAP threshold data were treated with *t*-testing to determine statistically significant differences in thresholds between groups. The DPOAE data were treated with a repeated-measures ANOVA to determine significant differences in responses before and after noise exposure. Spearman correlation coefficient was computed to assess how well hair cell loss (cytocochleograms) correlated with CAP thresholds and DPOAE recordings. Furthermore, Spearman correlation coefficient was also used to assess correlation between CAP thresholds and DPOAE responses. A *P*-value <0.05 was accepted as statistically significant for all computations.
